# Digital and Analog Detection of SARS-CoV-2 Nucleocapsid Protein via an Upconversion-Linked Immunosorbent Assay

**DOI:** 10.1021/acs.analchem.2c05670

**Published:** 2023-02-27

**Authors:** Julian C. Brandmeier, Natalia Jurga, Tomasz Grzyb, Antonín Hlaváček, Radka Obořilová, Petr Skládal, Zdeněk Farka, Hans H. Gorris

**Affiliations:** 1 Department of Biochemistry, Faculty of Science, Masaryk University, 625 00 Brno, Czech Republic; 2 Institute of Analytical Chemistry, Chemo- and Biosensors, University of Regensburg, 93053 Regensburg, Germany; 3 Department of Rare Earths, Faculty of Chemistry, Adam Mickiewicz University, Poznań, 61614 Poznań, Poland; 4 Institute of Analytical Chemistry of the Czech Academy of Sciences, 602 00 Brno, Czech Republic; 5 CEITEC − Central European Institute of Technology, Masaryk University, 625 00 Brno, Czech Republic

## Abstract

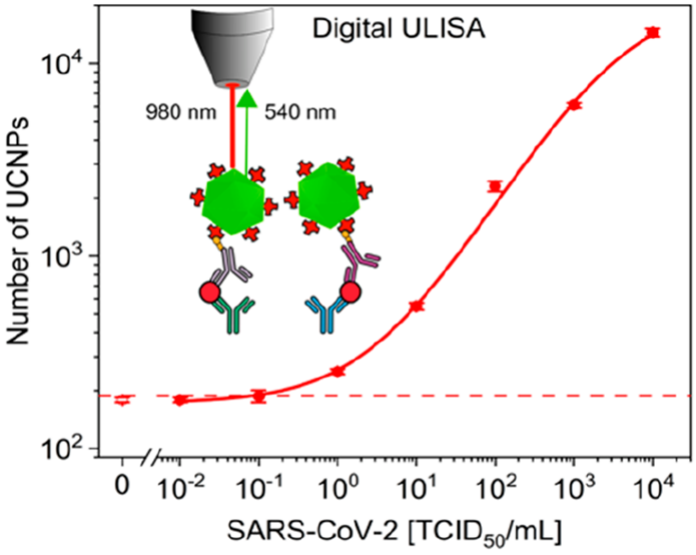

The COVID-19 crisis requires fast and highly sensitive tests for the early stage detection of the SARS-CoV-2 virus. For detecting the nucleocapsid protein (N protein), the most abundant viral antigen, we have employed upconversion nanoparticles that emit short-wavelength light under near-infrared excitation (976 nm). The anti-Stokes emission avoids autofluorescence and light scattering and thus enables measurements without optical background interference. The sandwich upconversion-linked immunosorbent assay (ULISA) can be operated both in a conventional analog mode and in a digital mode based on counting individual immune complexes. We have investigated how different antibody combinations affect the detection of the wildtype N protein and the detection of SARS-CoV-2 (alpha variant) in lysed culture fluid via the N protein. The ULISA yielded a limit of detection (LOD) of 1.3 pg/mL (27 fM) for N protein detection independent of the analog or digital readout, which is approximately 3 orders of magnitude more sensitive than conventional enzyme-linked immunosorbent assays or commercial lateral flow assays for home testing. In the case of SARS-CoV-2, the digital ULISA additionally improved the LOD by a factor of 10 compared to the analog readout.

## 1 Introduction

During the last three years of the COVID-19 pandemic, testing, social distancing, and finally vaccination have been the key factors in keeping the pandemic under control.^1^ In particular testing has been essential to identify asymptomatic individuals, whose contribution to virus transmission was largely underestimated at the beginning.^2^ Depending on the analyte, three types of SARS-CoV-2 assays can be distinguished: (1) Viral RNA tests based on PCR amplification are the most sensitive, but they have long turnaround times and are relatively expensive.^3^ (2) Serological tests detect whether a person has raised antibodies against SARS-CoV-2. As there is a lag time between an infection and an immune response, however, such assays are not amenable to early stage disease diagnosis. (3) Viral antigen tests are fast, cheap, and suitable for point-of-care testing, but they are typically less sensitive than PCR.^4^

The nucleocapsid protein (N protein) is the most abundant protein antigen in SARS-CoV-2 and shows lower mutation rates among different variants compared to the spike protein.^5^ As these features enable more sensitive measurements and a more reliable detection of different virus variants by the same antibodies, the N protein is commonly used as a target antigen in microtiter-plate enzyme-linked immunoassays (ELISA) and lateral flow immunoassays (LFA) intended for point-of-care testing.^6^ Various other assay formats and detection schemes for the diagnosis of SARS-CoV-2 have been reviewed recently.^7,8^

**Figure 1:**
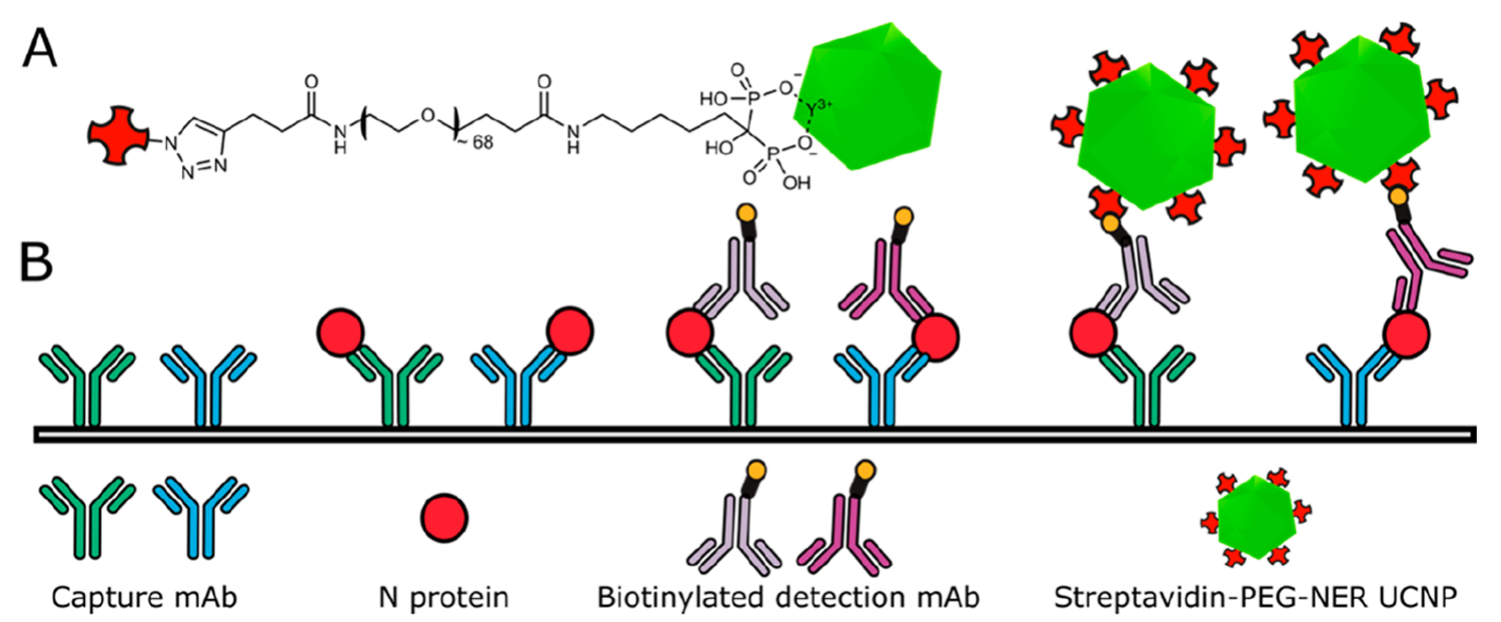
Detection of SARS-CoV-2 N protein. (A) UCNP label: Alkyne-PEG-neridronate strongly binds via two phosphonate groups to surface lanthanide ions of UCNPs, and a click reaction binds the conjugate to azide-modified streptavidin. (B) Scheme of sandwich ULISA: A microtiter plate is coated with two monoclonal antibodies that capture the N protein. Then, two biotinylated detection antibodies bind to the N protein. The sandwich immune complex is finally detected by using the UCNP label.

The optical readout of an enzymatic product in standard ELISAs or of colloidal gold in LFAs, however, is affected by optical background interference. By contrast, photon-upconversion nanoparticles (UCNP) emit shorter-wavelength light under near-infrared excitation (anti-Stokes emission) and thus eliminate optical background interference due to autofluorescence and light scattering.^9,10^ Consequently, immunoassays using UCNPs as a detection label (ULISA) have the potential to be >100-fold more sensitive compared to ELISA^11^ and LFA^12^ if nonspecific binding is efficiently avoided. Therefore, we developed water-dispersible and highly homogeneous UCNP labels that show a very low degree of nonspecific binding by employing a ligand exchange reaction with a neridronate poly(ethylene glycol) (PEG) conjugate (Figure 1A).^13^

The absence of optical background interference enables detecting and counting single UCNP-labeled immune complexes (digital mode) using a modified wide-field epiluminescence microscope.^14^ The digital ULISA is, in principle, not affected by variations in nanoparticle brightness (as long as they are bright enough for an unambiguous detection), particle aggregation, and instrumental background.^15^ We found, however, that the digital readout did not always result in a higher sensitivity compared to the conventional analog readout. While the detection of the cancer marker prostate-specific antigen (PSA) was 16-fold improved by using the digital readout,^11^ no significant improvement of the sensitivity was observed for the detection of human cardiac troponin I (cTnI), the most important marker of myocardial infarction.^16^ These experiments revealed that the sizes of UCNPs did not influence the assay sensitivity in the buffer but had a strong effect when plasma was used. For the detection of SARS-CoV-2, a UCNP-based test for viral oligonucleotides was reported,^17^ and a UCNP-based antigen test awaits market introduction.^18^ However, no original research report has been published, yet.

Here, we present a microtiter-based sandwich ULISA (Figure 1B) for the detection of N protein and SARS-CoV-2 and compare it to a conventional ELISA.^19^ The ULISA can be operated both in the analog and digital mode. Our earlier studies indicated that the digital readout is a necessary but not sufficient condition to achieve the highest possible assay sensitivity.^15^ The higher the antibody affinity is, the higher is the potential conferred by the digital readout. This is also in line with an earlier report that the LOD of the digital ELISA strongly depends on the antibody affinities.^20^ We have thus investigated the effect of different antibody combinations on the assay performance.

## 2 Materials and Methods

### 2.1 Reagents and Buffers

Recombinant SARS-CoV-2 N protein (full-length wildtype protein (GenBank: QHD43423.2) including C-terminal GS linker and His10-tag, *M*_W_ 47.1 kDa) and monoclonal anti-N protein antibody (mAb) clones C518, C524, C706, and C715 that bind to the N-terminal part (N47-A173) of the N protein were purchased from HyTest (Turku, Finland). Antibodies were characterized by surface plasmon resonance (SPR) and biotinylated as described in the Supporting Information. SuperBlock in Tris-buffered saline (TBS) was obtained from Thermo Fisher (Waltham, MA, USA), streptavidin-conjugated horseradish peroxidase (SA-HRP) from Abcam (Cambridge, UK), and TMB-Complete 2 substrate solution from TestLine Clinical Diagnostics (Brno, Czech Republic). Heat-inactivated SARS-CoV-2 culture fluid (alpha variant B.1.1.7, isolate USA/CA_CDC_5574/2020) was purchased from ZeptoMetrix (Buffalo, NY, USA) and used in a laboratory meeting BSL-2 standards.

Buffers were prepared using double-distilled water filtered through a 0.22-μm membrane (Magna Nylon, GVS, Zola Predosa, Italy). Buffers for the dilution of reagents included phosphate buffer (PB; 50 mM NaH_2_PO_4_/Na_2_HPO_4_, pH 7.4), phosphate-buffered saline (PBS; PB with 150 mM NaCl), and Tris-buffered saline (TBS; 50 mM Tris, 150 mM NaCl, pH 7.5). Coating buffer consisted of 50 mM NaHCO_3_/Na_2_CO_3_, 0.05% NaN_3_, and pH 9.6. Furthermore, washing buffer (50 mM Tris, 5 mM CaCl_2_, 0.05% Tween 20, pH 7.5), blocking buffer (10% SuperBlock in TBS, 1 mM KF, 0.05% NaN_3_, pH 7.5), and Tris assay buffer (10% SuperBlock in TBS, 1 mM KF, 0.05% Tween 20, 0.05% PEG, 0.05% NaN_3_, pH 7.5) were used. KF increases the stability of UCNPs in diluted aqueous dispersions.^21^ For the ELISA, the same buffers were prepared without NaN_3_ to avoid interference with the enzymatic activity of horseradish peroxidase.

Two commercial buffers for the lysis of SARS-CoV-2 capsids were obtained from Hangzhou Singclean Medical Products (Zhejiang, China) and Lotus NL (Den Haag, The Netherlands), denoted as “Lysis-Sing” and “Lysis-Lotus”, respectively. Additionally, the “Lysis-Guan” buffer^22^ containing guanidinium thiocyanate as a chaotropic reagent and Triton X-100 as a detergent and the “Lysis-X” buffer^23^ containing only Triton X-100 were prepared as described in the Supporting Information.

### 2.2 Preparation and Characterization of UCNP Labels

The syntheses of core/shell UCNPs (NaYF_4_: 18% Yb, 2% Er/NaYF_4_, 58 nm in diameter) and the alkyne-polyethylene(glycol)-neridronate linker (alkyne-PEG-ner) are described in the Supporting Information. For the preparation of SA-PEG-UCNP labels, 311 μL (10 mg) of UCNPs dispersed in cyclohexane was mixed with the same volume of 200 mM aqueous HCl and incubated for 30 min at 38 °C under shaking. The solution was then sonicated for 15 min to remove oleic acid from the UCNP surface and mediate a phase transfer from cyclohexane to water. The lower HCl phase was taken and added to an approximately 2-fold excess of acetone, which led to the precipitation of UCNPs. After centrifugation at 1000*g* for 20 min, the UCNP pellet was redispersed in 500 μL of water and sonicated for 5 min. Then, 2 mg of the linker dissolved in 500 μL of water was added and shaken overnight at 38 °C. Excess amounts of linker were removed by dialysis of the UCNP conjugates in a Float-A-Lyzer G2 dialysis device (100 kDa *M*_W_ cutoff, Fisher Scientific, Waltham, MA, USA) for 72 h at 4 °C against 4 L of 1 mM KF in water, which was exchanged nine times.

The UCNP conjugates were functionalized with streptavidin using a click reaction. Tris-HCl (375 mM, pH 7.5; 100 μL) and an aqueous solution of CuSO_4_ (25 mM; 10 μL) were added to 10 mg of alkyne-PEG-ner UCNPs dispersed in 1.4 mL of water. After purging the mixture for 45 min with argon, 100 μL of streptavidin azide (1 mg/mL) was added, and the mixture was purged for another 10 min. The click reaction was started by adding 20 μL of 100 mM sodium ascorbate in water. The dispersion was purged for 40 min with argon and then dialyzed for 72 h at 4 °C in a Float-A-Lyzer G2 dialysis device (100 kDa *M*_W_ cutoff) against 4 L of a dialysis buffer (50 mM Tris, 0.05% NaN_3_, 1 mM KF, pH 7.5), which was exchanged nine times.

The UCNPs and their conjugates were characterized using transmission electron microscopy (TEM), dynamic light scattering (DLS), and emission spectroscopy under 976 nm excitation as described in the Supporting Information.

### 2.3 Release of N Protein from SARS-CoV-2 in Culture Fluid and Nasopharyngeal Swabs

The manufacturer provided the concentration of SARS-CoV-2 in heat-inactivated culture fluid as the median tissue culture infectious dose (TCID_50_/mL = 1.05 × 10^6^). The TCID_50_/mL was also used to indicate the concentration of all further SARS-CoV-2 dilutions. For releasing the N protein from the virus, one part of culture fluid was mixed with nine parts of Lysis-Sing, Lysis-Lotus, Lysis-Guan, or Lysis-X, respectively, incubated under rotation for 20 min at room temperature and then diluted in Tris assay buffer. The virus lysate was prepared just before the immunoassay experiments.

Nasopharyngeal virus samples were collected by using cotton swabs. For the resuspension and lysis of the virus, cotton swabs were immersed and rotated in a vial containing Lysis-Sing, which was included with the LFA test kit. The virus lysate was prepared and 10-fold diluted in Tris assay buffer just before the LFA or immunoassay experiments.

### 2.4 Lateral Flow Assays

COVID-19 rapid antigen tests based on colloidal gold as a detection label were purchased from local retail stores and employed for reference experiments. (1) LFAs from Joinstar Biomedical Technology (Zhejiang, China) were used for the detection of SARS-CoV-2 in culture fluid. Culture fluid samples were 10-fold diluted in the supplied lysis buffer, and all further steps were performed according to the manufacturer’s instructions. (2) LFAs from New Gene Bioengineering (Hangzhou, China) were used for the detection of a volunteer’s active corona infection. After the onset of the first corona-related symptoms, nasopharyngeal swabs were collected daily as described above and stored at −20 °C until further use. All further steps were performed according to the manufacturer’s instructions. LFAs were considered positive when both the test (T) line and the control (C) line were detectable by eye and negative when only the C line showed a signal.

### 2.5 Microtiter-Based Immunoassays

The initial steps of ELISA and ULISA were carried out in the same way except for the following differences: (1) Standard high-binding 96-well microtiter plates (Greiner, Austria) were used for ELISA, while high-binding 96-well plates with a thin bottom foil (μClear, Greiner) were used for ULISA to allow for the digital readout under the microscope. (2) The ELISA reagents did not contain NaN_3_.

A 96-well microtiter plate was coated with 100 μL of a mixture of two monoclonal anti-N protein antibodies (C715 and C518, each 0.5 μg/mL) in a coating buffer overnight at 4 °C. All subsequent steps were carried out at room temperature. The plate was washed four times with 200 μL of the washing buffer. The plate was blocked for 1 h with 150 μL of the blocking buffer, washed four times, and 100 μL of serial dilutions of either the recombinant N protein or the virus lysate in Tris assay buffer was added and incubated for 1 h. The microtiter plate was washed four times with 200 μL of the washing buffer and incubated for 1 h with 100 μL of a mixture containing two biotinylated anti-N protein antibodies (C706 and C524, each 0.5 μg/mL) in a Tris assay buffer. The microtiter plate was washed four times with 200 μL of the washing buffer before the protocol for ELISA and ULISA diverged:

ELISA: A streptavidin-HRP conjugate (100 μL, 0.03 μg/mL in Tris assay buffer) was added for 1 h. The plate was washed four times with 200 μL of the washing buffer, and 100 μL of TMB substrate solution was added. After 1 min, 1 M sulfuric acid was added to stop the signal development, and the absorbance at 450 nm was measured on a Synergy 2 plate reader (BioTek, Winooski, VT, USA). A four-parameter logistic function was used for data fitting (Origin 2020, OriginLab, Northampton, MA USA), and LODs were calculated by adding three times the standard deviation of the blank to the baseline of the regression curve.

ULISA: SA-PEG-UCNPs (100 μL, 6.5 μg/mL) were added for 1 h in Tris assay buffer. The plate was then washed four times (200 μL) and left to dry.

#### 2.5.1 Analog Readout of ULISA

An upconversion microtiter plate reader (UPCON, Labrox, Turku, Finland) equipped with a 976 nm laser excitation source was used for measuring the upconversion luminescence (UCL) of Er-doped UCNPs at 540 nm in units of counts per second (CPS).^13^ In each well, 8 × 8 points were raster-scanned with a distance of 100 μm and a signal integration time of 1 s. The 16 highest and 16 lowest values were discarded, and the mean value was calculated, providing the truncated average of the intensity in a single well. The plotted averages and standard deviations were determined from three independent wells. The data was fitted by a four-parameter function. LODs were obtained by adding three times the standard deviation of the blank to the baseline of the regression curve.

#### 2.5.2 Digital Readout of ULISA

An inverted wide-field epifluorescence microscope (Eclipse Ti, Nikon, Tokyo, Japan) was connected to a continuous-wave 976 nm laser diode (4 W, Wavespectrum, Tianjin, China) via a multimode optical fiber (105 μm fiber core, 0.22 NA, Wavespectrum) and a motorized TIRF/epifluorescence illuminator unit (Eclipse Ti-E, Nikon). The filter cube for the detection of Er^3+^-doped UCNPs consisted of a long-pass excitation filter (λ_cut-on_ = 830 nm, Schott, Mainz, Germany), a dichroic mirror (λ_cut-on_ = 875 nm, AHF Analysentechnik, Tübingen, Germany), and a band-pass filter (λ = 535 ± 70 nm, OD_980_ ≈ 6, Chroma, Bellows Falls, VT, USA). The images were acquired on an sCMOS camera (5.5 megapixel; Neo, Andor Technology, Belfast, UK) and a 100× objective (1.49 NA; CFI HP Apochromat TIRF, Nikon), which resulted in a power density of 640 W/cm^2^.^13^

In each well filled with 100 μL of D_2_O for heat dissipation of the NIR laser beam, nine wide-field images of 166 μm × 144 μm were taken with a 100-fold objective and an exposure time of 7 s. The images were analyzed using NIS Elements 4.5 (Nikon). The total number of UCNPs in the nine images was counted automatically. The average and standard deviation were calculated from three wells, and the data were fitted using a four-parameter logistic function. LODs were obtained by adding three times the standard deviation of the blank to the baseline of the regression curve.

## 3 Results and Discussion

### 3.1 Optimization of Antibody Combinations for the Detection of Wildtype N Protein

The sensitivity of an immunoassay does not only depend on the assay design and labeling (Figures S1–S3) but also on the selection and combination of high-affinity antibodies, which need to be optimized for each analyte.^16^ The manufacturer of the monoclonal anti-N protein antibodies recommended the pairwise combination of two capture and two detection antibodies (2 + 2) for LFAs, which increases the likelihood of efficiently recognizing different variants (in this study, the wildtype virus and the alpha variant).^24^ They further tested and confirmed that the antibodies are not cross-reactive with other respiratory viruses including seasonal coronaviruses, which is important to prevent false-positive results.^25^

**Figure 2:**
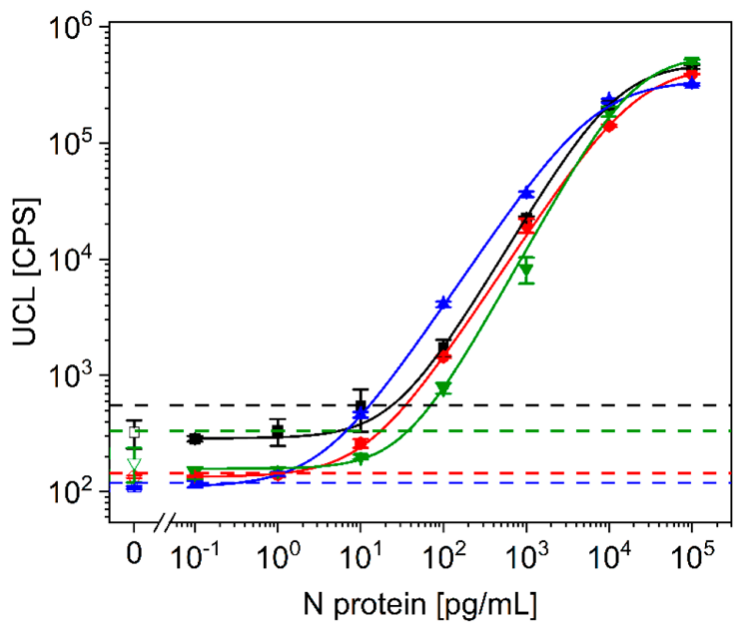
Calibration curves of the analog ULISA for the detection of wildtype N protein using different combinations of capture (c.a.) and biotinylated detection antibodies (d.a.). Black curve: c.a. 706, 524; d.a. 518, 715 (LOD 24 pg/mL). Green curve: c.a. 518, 706; d.a. 524, 715 (LOD 37 pg/mL). Red curve: c.a. 524, 715; d.a. 518, 706 (LOD 1.2 pg/mL). Blue curve: c.a. 518, 715; d.a. 524, 706 (LOD 0.33 pg/mL). The error bars represent the standard deviations of three replicate measurements. The hatched lines indicate three times the standard deviation of the background signal above the baseline of the regression curve.

We tested all possible 2 + 2 combinations of four mAbs for the detection of recombinant wildtype N protein (Figure 2). The respective detection antibodies were biotinylated for subsequent binding of streptavidin-UCNP labels. These antibody combinations resulted in up to 100-fold differences in the LOD, which was strongly, but not only, dependent on the degree of nonspecific binding (baseline of the regression curve). The combination of C518 and C715 as capture antibodies and biotinylated C524 and C706 as detection antibodies resulted in the lowest LOD (0.33 pg/mL; blue curve in Figure 2).

We discussed earlier that the association rates (*k*_on_) of antibody binding are more relevant for the performance of immunoassay since the dissociation rates (*k*_off_) are diminished by surface retention at the microtiter plate.^15^ SPR measurements showed that the two capture antibodies C715 and C518 had higher relative *k*_on_ rates than the detection antibodies C524 and C706 (Figure S2), which indicates that the capture efficiency is more strongly dependent on the antibody affinities than the detection efficiency. This optimal antibody combination was used in all further experiments.

**Figure 3:**
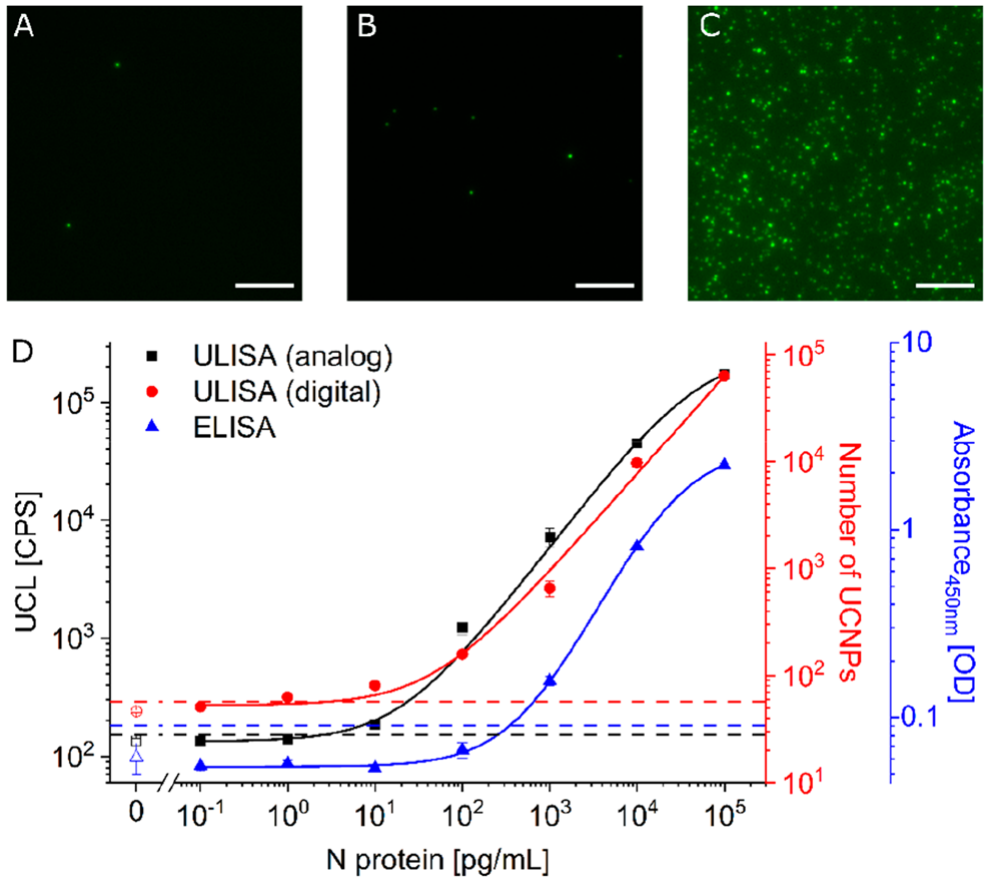
(A–C) Wide-field upconversion microscopy image sections (50 μm × 50 μm, scale bar: 10 μm) of the digital ULISA showing wildtype N protein concentrations of (A) 0 pg/mL, (B) 1000 pg/mL, and (C) 100,000 pg/mL. (D) Calibration curves of analog ULISA (black, LOD: 1.4 pg/mL), digital ULISA (red, LOD: 2.7 pg/mL), and ELISA (blue, LOD: 347 pg/mL). The error bars show the standard deviation of three replicate measurements. The hatched lines indicate three times the standard deviation of the background signal above the baseline of the regression curve.

Under the same experimental conditions, we replaced the streptavidin-UCNP label by streptavidin-conjugated horseradish peroxidase to implement a conventional microtiter plate ELISA as a reference method. The ELISA was approximately 1000-fold less sensitive (LOD: 347 pg/mL; Figure 3) than the ULISA. The ELISA is also more laborious because it requires two additional steps for adding TMB substrate solution and stopping solution, which prolong the assay protocol and time.

### 3.2 Comparison of Analog and Digital Readouts

The ULISA and ELISA measurements described in the previous section are based on signal integration within the entire detection area, which is denoted as an analog readout. By contrast, so-called digital immunoassays rely on counting individual immune complexes. Enzyme labels generate large numbers of product molecules that typically diffuse within the whole volume of a microtiter plate well and, thus, are not amenable to a digital readout unless product diffusion is efficiently prevented. For example, a digital ELISA has been implemented by separating the diffusion volume of thousands of enzyme-labeled immune complexes in large arrays of femtoliter-sized reaction wells.^26^ The digital ELISA achieved an LOD of 0.02 pg/mL for the detection of N protein,^20^ which is more than 10 times lower than the LOD obtained with the ULISA utilizing the best antibody combination. The authors noted that the LOD of the digital ELISA mainly depended on differences in the affinities of capture and detection antibodies because there was a wide range of LODs when measuring other SARS-CoV-2 antigen concentrations (spike protein: 70 pg/mL; spike protein subunit S1: 5 pg/mL).^20^

In the ULISA, however, individual immune complexes are directly linked to signal-generating UCNP labels, which can be counted as diffraction-limited spots in a conventional 96-well microtiter plate format under a wide-field upconversion microscope. Figure 3 shows examples of microscope images taken for the digital ULISA and the calibration curves of all three types of immunoassays.

Nonspecific binding of the UCNP label is detrimental for both the analog and the digital readout. Thus, we have optimized blocking conditions and the surface architecture of UCNPs to reduce nonspecific binding as efficiently as possible. Figure 3A shows a representative image of the blank sample with two nonspecific binding events, which adds up to 46 nonspecific binding events in the total area of nine images (0.2 mm^2^). With this number of counting events, the Poisson noise is 15%, which is larger than the variation between repeated measurements (experimental error: 3%, SI Table 1).

Both the analog and digital readouts benefit from the detection of UCNP labels without optical background interference, which explains the much higher sensitivity of the ULISA compared to the ELISA. Compared to the analog readout, the digital ULISA did not further improve the LOD, which we also observed earlier when developing a ULISA for the detection of troponin.^16^ Independent of the analog or digital readout, however, the detection of the N protein was 5-fold more sensitive than the detection of troponin, which confirms the role of the antibody affinity and the importance of finding the best antibody combination to achieve an optimal assay performance.

### 3.3 Analysis of SARS-CoV-2 in Cell Culture Fluid and in Nasopharyngeal Swabs

The N protein is, in principle, the optimal antigen for implementing COVID-19 immunoassays because it is the most abundant viral protein. This antigen, however, is not exposed on the viral surface and has to be released from the virus interior to be detectable. When testing different buffer compositions for the lysis of SARS-CoV-2 in culture fluid, we found that the lysis buffers strongly influenced the sensitivity of virus detection by the ULISA (Figure S4A). Among the two commercial buffers recommended for the use in LFA antigen tests, Lysis-Sing led to the most efficient release of the N protein and resulted in an LOD of 2 TCID_50_/mL, whereas Lysis-X led to a 60-fold worse performance. The lysis buffers prepared according to the protocols in the literature (Lysis-Guan and Lysis-X) were originally developed for releasing RNA from the virus capsids and PCR detection and resulted in an intermediate performance between the commercial buffers. When we preincubated the recombinant N protein for 20 min either with the optimal buffer Lysis-Sing or with Tris assay buffer only, these samples showed the same ULISA results (Figure S4B), and no interference of the lysis buffer with the immunoassay components was observable. A commercial rapid antigen test was positive too, even though at relatively high virus concentrations of >1000 TCID_50_/mL (Figure S5). Thus, we used Lysis-Sing for all further virus measurements.

**Figure 4:**
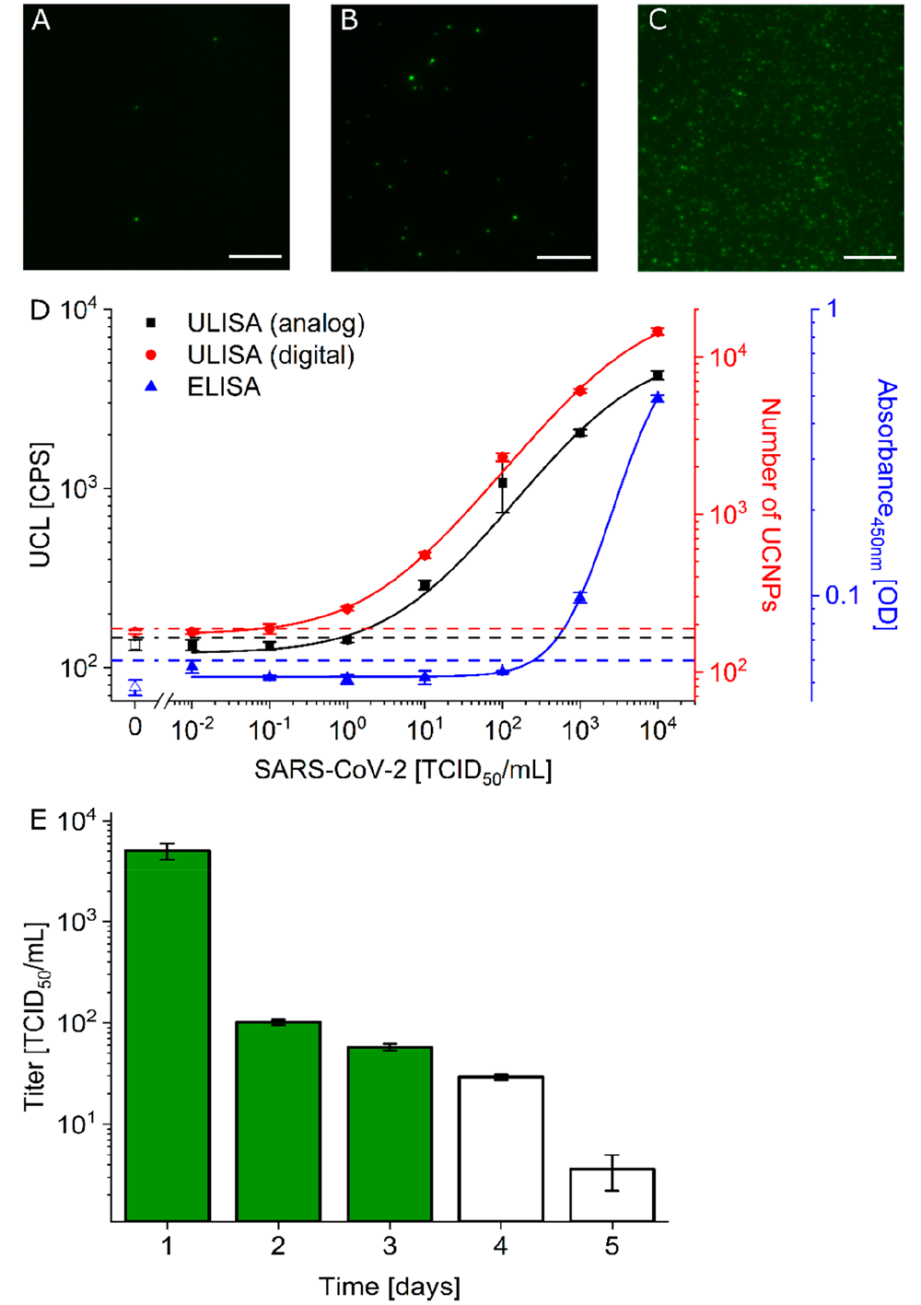
(A–C) Wide-field upconversion microscopy image sections (50 μm × 50 μm, scale bar: 10 μm) of the digital ULISA showing SARS-CoV-2 (alpha variant) concentrations of (A) 0 TCID_50_/mL, (B) 10^3^ TCID_50_/mL, and (C) 10^5^ TCID_50_/mL. (D) Calibration curves of the analog ULISA (black, LOD: 0.8 TCID_50_/mL), digital ULISA (red, LOD: 0.08 TCID_50_/mL), and ELISA (blue, LOD: 225 TCID_50_/mL) for the detection of SARS-CoV-2. (E) Time course of virus load in nasopharyngeal swabs after the onset of COVID-19-related symptoms (day 0) as determined by the analog ULISA (based on black calibration curve in 4D). Full bars indicate that additionally the reference LFA was positive and empty bars that the LFA was negative. Error bars show the standard deviation of three replicate measurements.

Figure 4 shows that the ULISA measured N protein concentrations in the virus lysate (alpha variant) with a 2000-fold lower LOD than the ELISA, which is comparable to the difference observed in the detection of the recombinant N protein (Figure 3). In the case of the virus lysate, however, the digital readout further improved the virus detection by 1 order of magnitude (LOD: 0.08 TCID_50_/mL). The antibody manufacturer reported that the antibodies have a higher specificity for the alpha variant of the N protein containing four amino acid mutations D3L/R203K/G204R/S235F (located outside the epitope binding regions N47-A173 of the antibodies) compared to the full-length wildtype N protein equipped with a C-terminal GS linker and a His10-tag.^24^ This result supports our initial hypothesis that a strong antigen–antibody interaction is required in the first place before the digital readout can further improve the ULISA sensitivity.

Next, we measured the virus load in nasal swabs during an active infection with SARS-CoV-2, which was confirmed by parallel LFA measurements. Nasopharyngeal swabs were collected using cotton swabs and processed in Lysis-Sing 1 day after the first corona-related symptoms. Figure 4E shows the time course of convalescence. The virus load was highest on day 1 and gradually decreased in the following days. While the ULISA showed a clearly positive response (UCL) after 5 days (190 CPS vs background of 133 CPS), the LFA was already negative on days four and five (Figure S6). The higher sensitivity of the ULISA thus enables the surveillance of an infection over a longer time than commercial home tests. Additionally, quantitative information on the virus load can be obtained, while the LFA only allows for a yes/no decision on an infection.

### 3.4 Comparison of ULISA with Other SARS-CoV-2 Tests

Table 1 summaries the LODs obtained by ULISA, ELISA, and LFA for the detection of both N protein and SARS-CoV-2 isolated from culture fluid. Compared to our assay results, standard electrochemical immunoassays feature similar sensitivities (LOD: 227 pg/mL)^27^ as the ELISA. By contrast, the ULISA is more comparable to an immunoassay based on electroluminescence with an additional enhancement step (MSD S-PLEX SARS-CoV-2 N assay kit),^28^ which was denoted as ultrasensitive (LOD: 0.16 pg/mL). The SIMOA platform, which includes an intrinsic preconcentration step on magnetic beads, enables the most sensitive N protein detection reported so far (LOD: 0.02 pg/mL).^20,29^ As mentioned earlier, however, it should be noted that the LODs also depend on antibody affinities and do not purely reflect the performance of the assay platform.

**Table 1:** LODs of Immunoassays for Detection of N protein and SARS-CoV-2 in Virus Lysates

Method	N protein (pg/mL)	SARS-CoV-2 (TCID_50_/mL)
Analog ULISA	1.4	0.8
Digital ULISA	2.7	0.08
ELISA	347	225
Commercial LFA	n.d.^a^	>1000

a n.d.: not determined.

The comparison of different assay platforms based on the TCID_50_/mL obtained with different virus preparations is even more difficult because the TCID_50_ strongly depends on the virus inactivation process. As shown in Figure S4, the LOD varies additionally depending on how efficient the N protein is released from the virus. A capacitive biosensor using vertically paired electrodes reported a similar LOD as the ELISA (LOD: 147 TCID_50_/mL).^30^ Finally, an LFA for N protein detection was similar in sensitivity (650 pg/mL or 3030 pg depending on the source of N protein)^31^ as the LFAs used for our reference experiments and enabled the detection of 4 TCID_50_/swab, which was equivalent to 25,000 virus copies/swab.^32^

RT-PCR is typically more time consuming than optical detection assays and in principle can amplify a single RNA strand to a measurable signal, which may return a positive test result long after an individual has ceased to be infectious. The ULISA fills a niche because it is more sensitive than available LFAs but less sensitive than RT-PCR and thus may report on the acute status of patient infectivity more precisely.

## 4 Conclusions

Consistent with our earlier studies on PSA^11,33^ and troponin,^16^ the ULISA improved the detection of both N protein and SARS-CoV-2 by about 3 orders of magnitude. While the sensitivity of wildtype N protein detection was independent of the analog or digital readout, the digital ULISA improved the detection of SARS-CoV-2 in virus lysates further by a factor of 10. These different performance characteristics may result from the stronger antibody–antigen interaction of the alpha variant N protein compared to the recombinant wildtype N protein, which supports our hypothesis that a strong antigen–antibody interaction is a first requirement before the digital readout can further boost the ULISA sensitivity. However, even in the analog mode, the ULISA clearly outperforms the ELISA not only in terms of a much lower LOD, but also in terms of a wider signal-to-background ratio and fewer assay steps. The higher sensitivity of the ULISA enables early diagnosis and thus lowers the probability of further spreading an infection. Furthermore, the ULISA is relatively easy to perform and can be adapted to other diagnostically relevant biomarkers.

## Supporting Information Available

Synthesis of UCNPs; synthesis of alkyne-PEG-neridronate; characterization of UCNPs; surface plasmon resonance measurements (SPR) of antibody affinities; biotinylation of antibodies; wide-field epiluminescence microscopy; characterization of UCNPs by TEM, DLS, and spectroscopy; brightness distribution of single diffraction limited spots; preparation and performance of lysis buffers; results of LFA tests; and precision of digital ULISA (PDF)
