# Supplementary material for: Digital and Analog Detection of SARS-CoV-2 Nucleocapsid Protein via an Upconversion-Linked Immunosorbent Assay
Source: Anal Chem. 2023 Feb 27;95(10):4753–9. doi: 10.1021/acs.analchem.2c05670 (PMC10018451; doi:10.1021/acs.analchem.2c05670)
Supplement: Supplementary file 1 — ac2c05670_si_001.pdf [file ac2c05670_si_001.pdf]

# - Supporting Information -

Digital and Analog Detection of SARS-CoV-2 Nucleocapsid Protein via an Upconversion-Linked Immunosorbent Assay

Julian C. Brandmeier,[1,2] Natalia Jurga,[1,3] Tomasz Grzyb,[3] Antonín Hlaváček,[4] Radka Obořilová,[1] Petr Skládal,[1,5] Zdeněk Farka,[1,5] and Hans H. Gorris[1,*]

[1]Department of Biochemistry, Faculty of Science, Masaryk University, Brno, Czech Republic

[2]Institute of Analytical Chemistry, Chemo- and Biosensors, University of Regensburg, Regensburg, Germany

[3]Department of Rare Earths, Faculty of Chemistry, Adam Mickiewicz University, Poznań, Poland

[4]Institute of Analytical Chemistry of the Czech Academy of Sciences, Brno, Czech Republic

[5]CEITEC – Central European Institute of Technology, Masaryk University, Brno, Czech Republic

Additional methods

Synthesis of UCNPs

Synthesis of alkyne-PEG-neridronate

Characterization of UCNPs

Surface plasmon resonance (SPR) measurements of antibody affinities

Biotinylation of monoclonal antibodies

Preparation of buffers for the lysis of SARS-CoV-2

Wide-field epiluminescence microscopy

Supporting Figures

Supporting Figure S1. Characterization of UCNPs

Supporting Figure S2. SPR measurements of SARS-CoV-2 N protein

Supporting Figure S3. Brightness distribution of single diffraction limited spots

Supporting Figure S4. Performance of different lysis buffers

Supporting Figure S5. Detection of SARS-CoV-2 in culture fluid using a commercial LFA

Supporting Figure S6**.** Detection of an active COVID-19 infection using a commercial LFA

Supporting Table

Supporting Table 1: Precision of the digital ULISA

Synthesis of UCNPs

$YCl_3 \times 6\ H_2O$ (874 mg, 2.88 mmol), $YbCl_3 \times 6\ H_2O$ (251 mg, 0.648 mmol), and $ErCl_3 \times 6\ H_2O$ (27.5 mg, 0.072 mmol) were dissolved in 30 mL of methanol and added into a 250-mL three-neck round-bottom flask containing 27 mL (24.2 g) of oleic acid and 63 mL (49.7 g) of 1-octadecene. The solution was heated to 170 °C under a protective nitrogen atmosphere until all volatile liquids were evaporated (ca. 60 min). After the temperature had decreased to 50 °C, the nitrogen atmosphere was disconnected, and a solution of 533 mg (14.4 mmol) of NH_4F and 360 mg (9 mmol) of NaOH in 30 mL of methanol was added under intense stirring. The nitrogen atmosphere was reconnected, and the solution was stirred for 30 min. The temperature was carefully increased up to 150 °C, avoiding extensive boiling to ensure the evaporation of methanol. After that, the solution was rapidly heated at the rate of ca. 10 °C/min. At 290 °C, the heating was carefully adjusted to 300 °C within one or two minutes. The flask was kept under nitrogen flow at 300 °C (± 4 °C) for 90 min and then let to cool down to RT. The emerging UCNPs were precipitated by adding 180 mL of propan-2-ol and collected by centrifugation (1,000 g, 10 min). The precipitate was washed with 90 mL of methanol, centrifuged (1,000 g, 10 min) and redispersed in 20 mL of cyclohexane. After adding 100 mL of methanol, the UCNPs precipitated rapidly without centrifugation. The precipitate was redispersed in 30 mL of cyclohexane and slowly centrifuged (50 g, 20 min) to separate solid compounds from the final UCNPs.

Under reflux, Y_2O_3 (1355 mg, 6.00 mmol), Yb_2O_3 (532 mg, 1.35 mmol) and Er_2O_3 (57.9 mg, 0.15 mmol) were dissolved in trifluoroacetic acid (12 mL) and water (12 mL) in a 250-mL three-necked flask. When dissolved, $NaHCO_3$ (1260 mg, 15.00 mmol) was added, releasing CO_2 bubbles and dissolving rapidly, resulting in a clear solution. After removing the condenser, excessive trifluoroacetic acid and water were evaporated by heating at 110 °C in a fume hood (overnight). The resulting white powder of trifluoroacetates was dissolved in oleic acid (45 mL, 40.3 g) and octadec-1-ene (45 mL, 35.5 g). This solution was diluted by 30 mL of methanol. The methanol together with oxygen and water were removed by heating at 110 °C under the N_2 atmosphere for 20 min, resulting in a precursor solution. The precursor solution was enclosed in the flask by silicon septa and kept under an inert atmosphere. To decrease the viscosity, the precursor solution was kept at an elevated temperature (ca. 50 °C), which facilitated its injection into the hot reaction mixture. The concentration of $Re(CF_3CO_2)_3$ in the precursor solution was 0.17 mmol/mL (Re for Y, Yb, Er in molar percentages 80%, 18% and 2.0%, respectively.

The nanoparticles were grown by gradually adding the precursor solution to the solution of seed nanoparticles. The dispersion of seed nanoparticles (205 mg) in cyclohexane was mixed with oleic acid (5.5 mL, 4.9 g), octadec-1-ene (17 mL, 13.4 g), and 20 mL of methanol in a 100-mL three-necked flask. The mixture was heated at 150 °C for ~30 min under the nitrogen atmosphere to remove oxygen and water. Then, the temperature was rapidly increased to 300 °C. Keeping this temperature, a syringe with a long needle was used to inject the precursor solution nine times (respective volumes: 3.5, 4.0, 4.6, 5.6, 6.2, 7.1, 8.3, 9.6 and 10.1 mL), each with a delay of 10 min. After this, the 100-mL flask was full, and the reaction mixture was transferred to a 250-mL three-necked flask together with 30 mL of methanol. The solution was heated to 150 °C for ~30 min under an inert nitrogen atmosphere to remove oxygen, methanol and water. The temperature was rapidly increased to 300 °C, and the 9-fold injection or precursor solution was repeated; the temperature was kept at 300 °C for 10 min, eventually preparing nanoparticles of the desired size (reaction mixture volume ~82 mL), and the synthesis continued without decreasing the temperature by growing the inert shell of $NaYF_4$.

Under reflux, Y_2O_3 (1694 mg, 7.50 mmol) was dissolved in trifluoroacetic acid (12 mL) and water (12 mL) in a 250-mL three-necked flask. When dissolved, $NaHCO_3$ (1260 mg, 15.00 mmol) was added, releasing CO_2 bubbles and dissolving rapidly to a clear solution. After removing the condenser, excessive trifluoroacetic acid and water were evaporated by heating at 110 °C in a fume hood (overnight). The resulting white powder of trifluoroacetates was dissolved in oleic acid (45 mL, 40.3 g) and octadec-1-ene (45 mL, 35.5 g). This solution was diluted by 30 mL of methanol. The methanol together with oxygen and water were removed by heating at 110 °C under an inert nitrogen atmosphere for 20 min, resulting in a precursor solution. The precursor solution was enclosed in the flask by a silicon septum and kept under an inert atmosphere. To decrease the viscosity, the precursor solution was held at ~50 °C, which facilitated its injection into the hot reaction mixture. The precursor solution contained 0.17 mmol/mL of $Y(CF_3CO_2)_3$.

The shell was grown by gradually adding the precursor solution to the hot solution of grown nanoparticles from the previous step. A calculated amount of precursor solution was repeatedly injected by a syringe with a long needle (120 mm) without decreasing the temperature. The three additions were of 8.0, 9.0, and 10.0 mL; the interval between the injections was 10 min. After the last injection, the temperature was kept at 300 °C for an additional 10 min, eventually preparing the desired nanoparticles (the volume of the reaction mixture was ~95 mL). Finally, the flask was cooled to room temperature. The resulting nanoparticles were precipitated by

adding propan-2-ol (190 mL) and collected by centrifugation (1,000 g, 10 min). The pellet was washed with methanol (109 mL), centrifuged (1,000 g, 10 min), and dispersed in cyclohexane (60 mL). After the last precipitation by methanol, the nanoparticles were dispersed in cyclohexane and slowly centrifuged (50 g, 20 min) to separate coarse particles from the final product.

Synthesis of alkyne-PEG-neridronate (alkyne-PEG-ner)

First, 30 mg of neridronate (Merck, Darmstadt, Germany) was dissolved in a mixture of 898 µL of PB (50 mM, pH 7.4) and 128 µL of 1 M NaOH under sonication. Then, 75 mg of Alkyne-PEG-NHS (α-*N*-hydroxysuccinimide-ω-alkyne polyethylene glycol, M_W 3000; Iris Biotech, Marktredwitz, Germany) was added and incubated overnight at 4 °C. The reaction mixture was dialyzed against 4 L of bidistilled H_2O in a Float-A-Lyzer G2 dialysis device (MW cut_{off} = 500–1000 Da, Fisher Scientific, Germany) at 4 °C for 96 hours; H_2O was exchanged 12 times. The purified, colorless Alkyne-PEG-ner was lyophilized (Alpha 1-2, Christ, Osterode am Harz, Germany) and stored at 4 °C.

Characterization of UCNPs

Oleic acid-coated UCNPs dispersed in 5 µL of cyclohexane were dispensed on a copper grid coated with a 12-nm continuous carbon foil. A paper tissue was used to remove excess amounts of fluid, and the grid was dried on air. TEM images were recorded on a Titan Themis (FEI, Czech Republic). SA-PEG-UCNPs dispersed in 2 µL of water were dispensed on a copper grid covered with a holey carbon film. A paper tissue was used to remove excess amounts of fluid, and the grid was dried on air. TEM images were recorded on a HT7700 TEM (Hitachi, Japan).

The hydrodynamic diameter of UCNPs was determined on a Zetasizer Nano (Malvern, UK) using dynamic light scattering (DLS). Dispersions of 187 µg/mL of oleic acid-capped UCNPs in cyclohexane and 325 µg/mL of SA-PEG-UCNPs in 50 mM Tris were analyzed.

The emission spectrum of 30 mg/mL of oleic acid-coated UCNPs dispersed in cyclohexane was recorded by a self-made epiluminescence detector equipped with a 979-nm laser module (400 mW) and an excitation intensity of ~200 W/cm^2.

Surface plasmon resonance (SPR) measurements of antibody affinities

An MP-SPR Navi 210A SPR system (Bionavis, Tampere, Finland) equipped with a 670-nm laser was used to scan the spectral angle between 58° and 78°. The shift of the SPR angle was determined using a centroid fitting function. mAbs C715, C518, C524 or C706, respectively,

were immobilized on a commercial CMD200M SPR chip coated with carboxymethyl dextran to measure the binding kinetics of the N protein label-free and in real-time.

The running buffer consisting of 10 mM HEPES (Carl Roth, Karlsruhe, Germany), 150 mM NaCl and 0.01% Tween20 (pH 7.4) was degassed before each measurement. The chip surface was rinsed continuously with running buffer at a flow rate of 20 µL/min until a stable SPR signal was reached. Then, the surface was activated by pumping 0.2 M EDC and 0.05 M NHS (1:1) in water into the channels for 10 min. The respective antibodies in 50 mM acetate buffer (pH 4.5) were pumped for 20 min into the measurement channel at a flow rate of 10 µL/min. No antibody was inserted into the control channel. Subsequently, the surface was blocked for 5 min by 1 M of an aqueous ethanolamine solution (pH 8). The N protein (concentrations of 0.1, 1, 10, 100, 1000 ng/mL) in running buffer was applied at a flow rate of 20 µL/min until a stable, high binding signal was reached. Finally, the surface was regenerated using 10-100 mM of HCl for 2 min.

Biotinylation of monoclonal antibodies

A solution of 5 mg/mL of NHS-LC-biotin (Merck/Sigma-Aldrich, St. Louis, MO USA) in dry DMF was prepared. A solution of 95.4 µg of mAb in PBS was mixed with 1.15 µL of NHS-LC-biotin, and PBS was added to obtain a final volume of 84.3 µL. After the solution had been shaken for 10 min, another 1.15 µL of NHS-LC-biotin was added, and the reaction mixture was shaken for 2 h at RT. The biotinylated antibody was purified six times via centrifugation (14,000 g, 20 min) using Amicon ultra centrifugal filters (MWCO 100 kDa, Merck KGaA, Darmstadt, Germany), transferred to PBS and stored at 4 °C at a concentration of 1 mg/mL.

Preparation of buffers for the lysis of SARS-CoV-2

Reagents: Tris-HCl (Carl Roth); guanidinium thiocyanate (Sigma Aldrich); $Na_2EDTA \times 2\ H_2O$ (Carl Roth); Triton X-100 (Sigma Aldrich)

Lysis-Guan[1] (contains guanidinium thiocyanate as a chaotropic reagent)

Tris-HCl (5 mM) was dissolved in bidistilled H_2O under continuous stirring and adjusted to pH 6.4 using 0.1 M NaOH. Guanidinium thiocyanate (5 M) was added under ultrasonication at 65 °C. In a separate flask, $Na_2EDTA \times 2\ H_2O$ (50 mmol) was mixed with bidistilled H_2O, and NaOH pellets were added under continuous stirring until the EDTA was completely dissolved. The EDTA solution was adjusted to pH 8.0 with 0.1 M NaOH, filtered, and finally added to the guanidinium thiocyanate solution to obtain a final concentration of 22 mM of EDTA. Triton X-100 (1.2 wt/v) was added to the solution under shaking until the solution was homogenous.

Lysis-X[2] (contains only Triton-X 100 as a detergent)

NaCl (150 mM) and Tris-HCl (10 mM) were dissolved in bidistilled H_2O under constant stirring. After adding Triton X-100 (0.25 %), the solution was homogenized under ultrasonication and adjusted to pH 7.4 using 0.1 M NaOH.

References:

(1) R. Boom, C. J. Sol, M. M. Salimans, C. L. Jansen, P. M. Wertheim-van Dillen, J. van der Noordaa (1990). Rapid and Simple Method for Purification of Nucleic Acids. *J. Clin. Microbiol. 28*, 495-503.

(2) K. Shatzkes, B. Teferedegne, H. Murata (2014). A Simple, Inexpensive Method for Preparing Cell Lysates Suitable for Downstream Reverse Transcription Quantitative PCR. *Sci. Rep. 4*, 4659.

Additional results

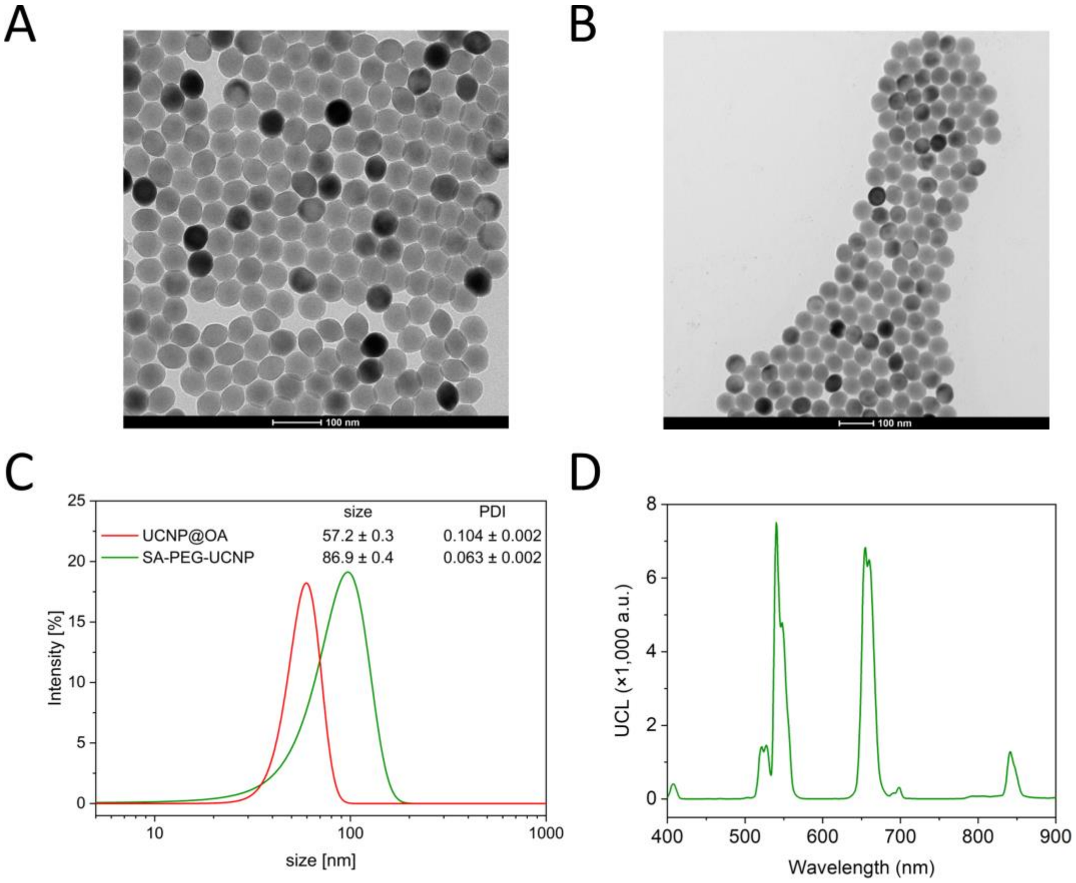

Supporting Figure S1. Characterization of UCNPs ($NaYF_4$: 18% Yb, 2% Er / $NaYF_4$, 58 nm in diameter): Transmission electron microscopy (TEM) images of (**A**) oleic acid-capped UCNPs and (**B**) SA-PEG-UCNPs, (**C**) dynamic light scattering (DLS), and (**D**) emission spectroscopy under 976-nm excitation.

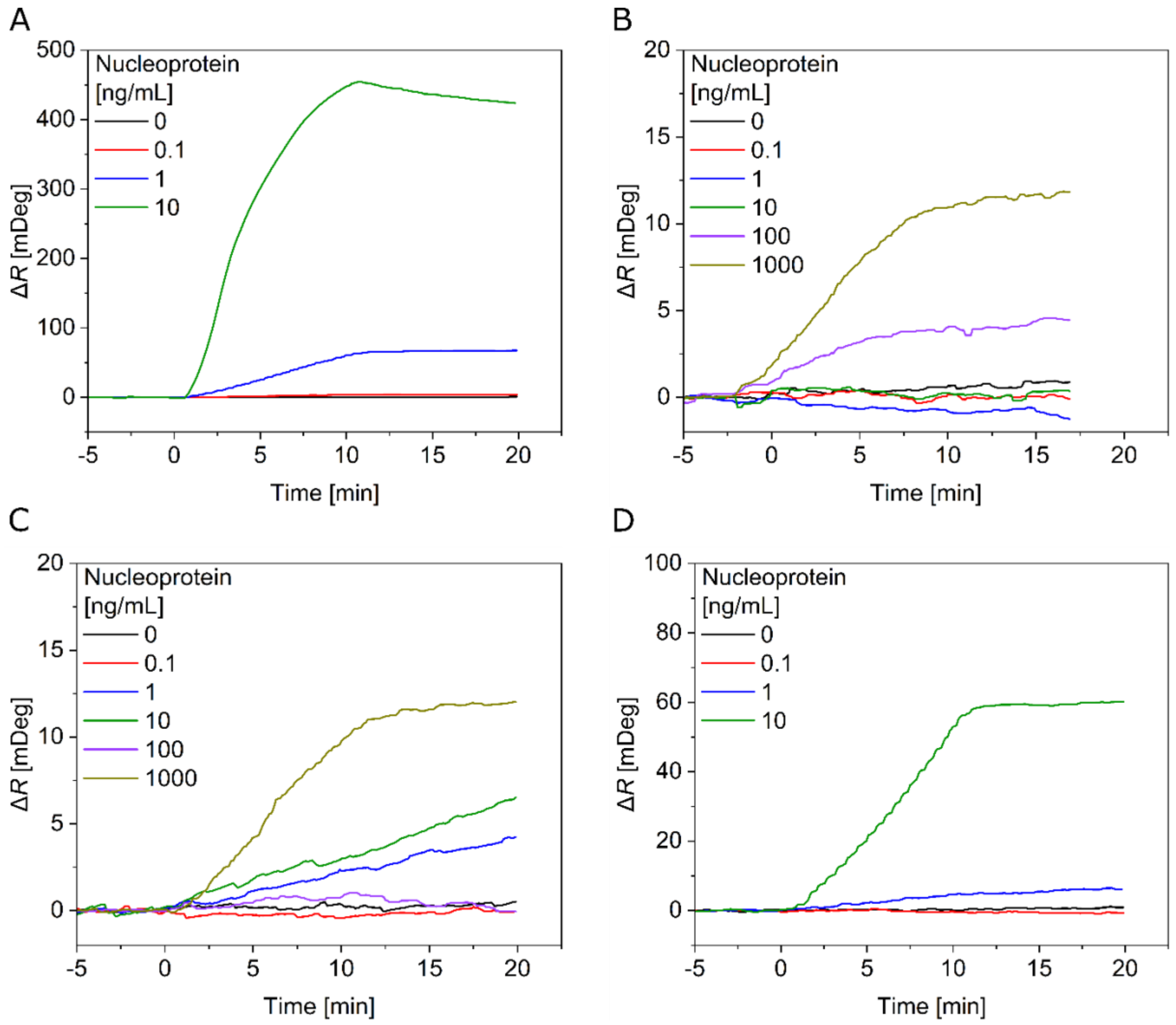

Supporting Figure S2. SPR measurements of N protein binding by surface-immobilized mAbs A) C518, B) C524, C) C706 and D) C715. mAbs C518 and C715 show strong signal increases already at N protein concentrations of 10 ng/mL, while mAbs C524 and C706 require 100-fold higher N protein concentrations (1000 ng/mL) to show a similar signal response.

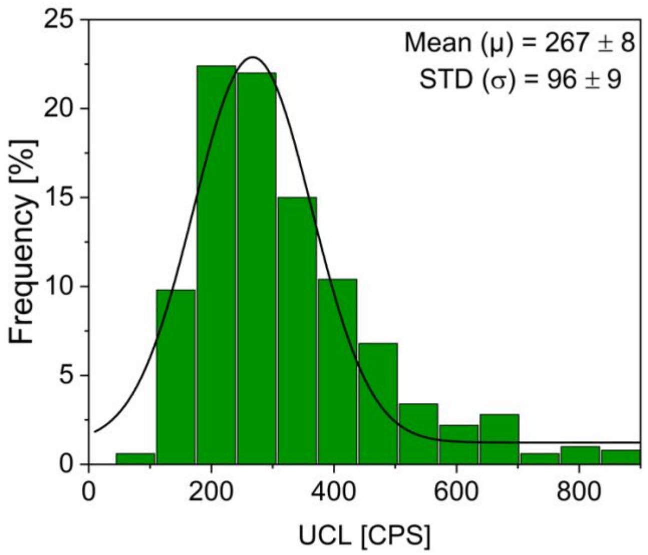

Supporting Figure S3. Brightness distribution of individual diffraction limited spots taken at a viral concentration of 10^3 $TCID_{50}$/mL under the wide-field upconversion microscope (Fig. 4).

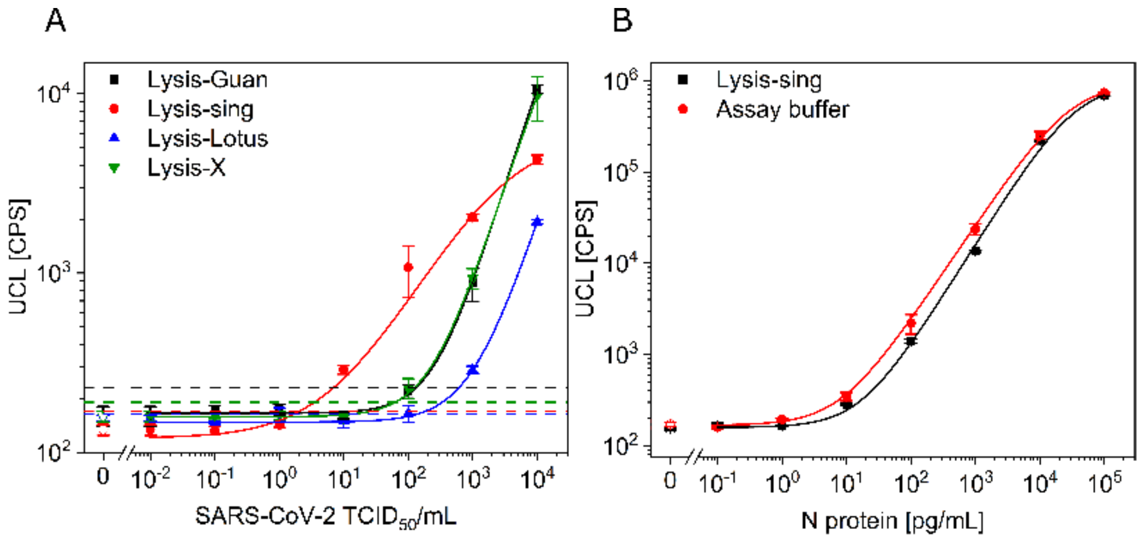

Supporting Figure S4. A) Influence of different lysis buffers on the detection of SARS-CoV-2. Culture fluid was tenfold diluted in lysis buffers and further diluted in Tris assay buffer ($LOD_{Lysis\text{-}Guan}$: 131.8 $TCID_{50}$/mL; $LOD_{Lysis\text{-}Sing}$: 2.0 $TCID_{50}$/mL; $LOD_{Lysis\text{-}Lotus}$: 138.2 $TCID_{50}$/mL, $LOD_{Lysis\text{-}X}$: 63.3 $TCID_{50}$/mL). **B)** Influence of the optimal lysis buffer Lys-Sing on the detection of recombinant wildtype N protein. The N protein was tenfold diluted in Lysis-Sing (LOD: 1.2 pg/mL) or Tris assay buffer (LOD: 1.4 pg/mL), respectively, and further diluted in Tris assay buffer. Error bars represent the standard deviation of three replicate measurements.

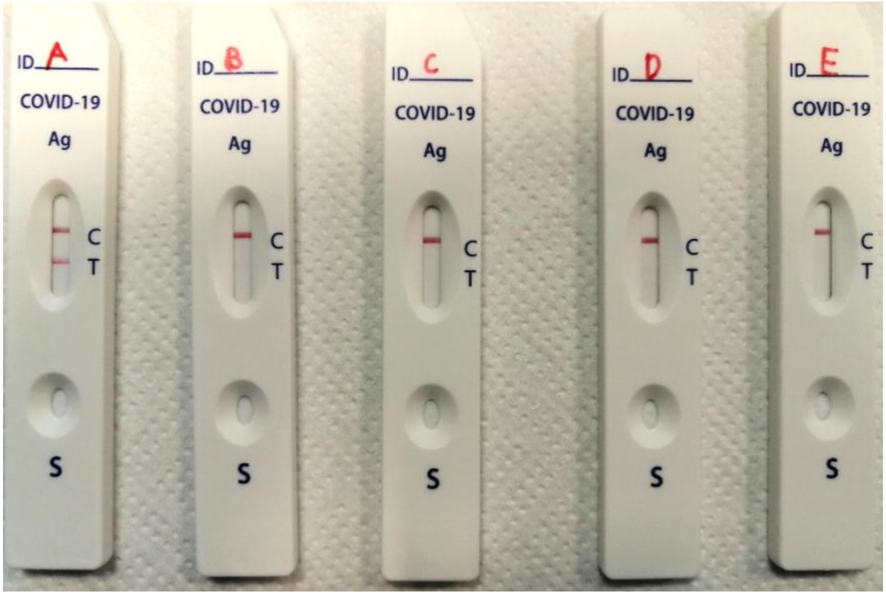

Supporting Figure S5. Detection of SARS-CoV-2 using a commercial LFA for self-testing (Joinstar Biomedical Technology). Culture fluid was incubated with the supplied lysis buffer and dilutions of (A) 10^5 $TCID_{50}$/mL, (B) 10^3 $TCID_{50}$/mL, (C) 10^1 $TCID_{50}$/mL, (D) 10^{-1} $TCID_{50}$/mL, and (E) 0 $TCID_{50}$/mL were dispensed on the sample application pad (S). Viral concentrations of 10^5 $TCID_{50}$/mL led to a positive test result (red signal in T line), whereas lower concentrations remained undetected (no signal in T line).

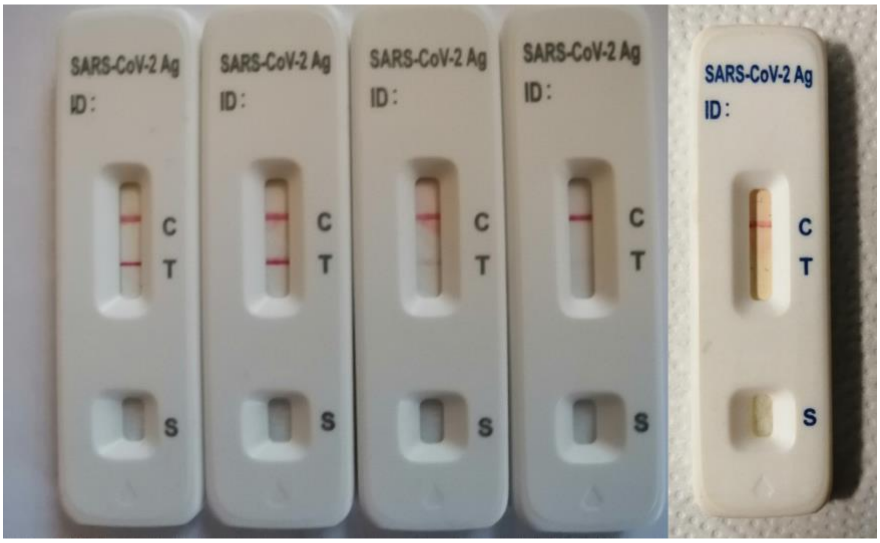

Supporting Figure S6. Detection of SARS-CoV-2 using a commercial LFA for self-testing (New Gene Bioengineering). Nasopharyngeal swabs were collected and analyzed from day 1 to day 5 (from left to right) after the onset of symptoms. The LFA was negative after day 3.

Supporting Table 1: Precision of the digital ULISA. The coefficient of variation (CV) was calculated by dividing the standard deviation of three wells by the average number of UCNPs per well. The Poisson noise was calculated by dividing the square root of the average number of UCNPs in an area of 0.2 cm^2 (9 images of 166 × 140 μm^2 combined) by the average number of UCNPs in that area ($\sqrt{n}/n$).

	Concentration	Average # of UCNPs	Experimental CV	Poisson noise
blank	0 pg/mL	46 ± 1	3.3%	14.7%
N protein (wildtype) (Figure 3)	0.1 pg/mL	51 ± 2	3.9%	14.0%
	1 pg/mL	70 ± 1	2.2%	12.0%
	10 pg/mL	92 ± 1	1.1%	10.4%
	100 pg/mL	160 ± 4	2.3%	8.0%
	1,000 pg/mL	690 ± 28	4.1%	3.8%
	10,000 pg/mL	9650 ± 28	9.0%	1.0%
	100,000 pg/mL	64000 ± 3800	5.9%	0.4%
SARS-CoV-2 (alpha) (Figure 4)	0.1 $TCID_{50}$/mL	178 ± 5	2.9%	7.5%
	1 $TCID_{50}$/mL	190 ± 13	7.4%	7.3%
	10 $TCID_{50}$/mL	250 ± 7	3.0%	6.3%
	100 $TCID_{50}$/mL	550 ± 23	4.3%	4.3%
	1,000 $TCID_{50}$/mL	2300 ± 140	6.0%	2.1%
	10,000 $TCID_{50}$/mL	6100 ± 170	2.8%	1.3%
	100,000 $TCID_{50}$/mL	14500 ± 690	4.7%	0.8%
